# Supplementary material for: Involving Children in Creating a Healthy Environment in Low Socioeconomic Position (SEP) Neighborhoods in The Netherlands: A Participatory Action Research (PAR) Project
Source: Int J Environ Res Public Health. 2021 Nov 19;18(22):12131. doi: 10.3390/ijerph182212131 (PMC8624284; doi:10.3390/ijerph182212131)
Supplement: Supplementary file 1 [file ijerph-18-12131-s001.zip › ijerph-1431450-supplementary.pdf]

Additional File S1. Overview of derived themes, conclusions, and actions.

	Themes	Conclusions	Actions
**Neighborhood A**	Garbage in public space	People dump their garbage in the hallways between houses.<br>Children are hindered to play outside because of broken glass in playground sand.	Making warning signs which say that everybody should throw away there garbage as they should.
	Destruction of playground equipment	Destruction (vandalism) of playground equipment by youth happens regularly.	There was no action undertaken, because this was not prioritized by the children.
	Nuisance	Some places in the neighborhood are not pleasant to visit, for example, because of loitering youths smoking weed.	There was no action undertaken, because this was not prioritized by the children.
	Variety in playground equipment	According to the children, there is little desirable playground equipment in the neighborhood. There is not much variety in equipment: there are no options for climbing, for example. The table tennis table is not being used because of sticky resin on the table.	Writing a letter to the local policymaker. In this letter the children asked for more variety in playground equipment in the neighborhood. The children were invited to think along in the design and decision on new equipment, after project MAPZ ended. The involved community worker facilitated this.

**Neighborhood B**	Playground equipment for older children	Most playground equipment is for very young children (0-8 y/o). The participating children in the project (8-12 y/o) find the equipment childish, and too small.	Recording a video to show policymakers that the current playground equipment is only fun for younger children and not challenging enough for older children. This video was shown to the policymakers after project MAPZ ended and a discussion followed. The involved community worker facilitated this. There was at the time not enough resources and priority to change any equipment in the neighborhood.
	Sedentary behavior/screen time	Many children use screens after school, which makes being physically active outside less fun.	A conversation was held to discuss this issue with other children and make them aware of this.
**Neighborhood C**	Garbage in public space	Because of insufficient garbage bins in the neighborhood, a lot of garbage is on the streets. This makes being physically active outside less attractive, according to the children.	The children set up the action 'Adopt a garbage bin'. They handed out flyers to people living in the neighborhood and asked them to adopt a garbage bin. This would entail emptying the bin when it's empty. There were no registrations from neighborhood residents when project MAPZ ended.

	Nature	Nature encourages being physically active outside, according to the children. The reason for this is that children like to be at places where there is nature.	There was no action undertaken, because the children were satisfied with the current situation.
	Behavior guidelines for the Johan Cruyff soccer court	Older children tell younger children they cannot play soccer with them. Children are cycling through the soccer field, which is annoying, according to the children.	Making a sign with behavior guidelines. This sign was hung up at the entrance of the soccer court.
	Opening hours of the rainbow playground	The rainbow playground is often closed.	Writing a letter to the playground manager/porter and inviting them over for a conversation.
